# Screening for adolescent alcohol and drug use in pediatrics: predictors and implications for practice and policy

**DOI:** 10.1186/1940-0640-7-S1-A15

**Published:** 2012-10-09

**Authors:** Stacy Sterling, Andrea Hessel, Charles Wibbelsman

**Affiliations:** 1 Kaiser Permanente Northern California Division of Research, Oakland, CA, USA; 2 Kaiser Permanente Northern California/San Francisco Medical Center, San Francisco, CA, USA

## 

We describe findings from a web survey of pediatric primary care providers (PCPs) and a pilot study of a screening, brief intervention, and referral to treatment (SBIRT) model of primary care-based adolescent behavioral health care. The survey (N = 437) examined PCP attitudes and knowledge, patient characteristics, and environmental influences, (e.g., mental health parity and medical marijuana laws). We examined how PCP, panel, and organizational characteristics influence screening practices. The pilot examined whether SBIRT versus usual care increased problem identification and specialty treatment rates and the feasibility of SBIRT in pediatric patients. Respondents were less concerned about alcohol than other drug use, rated alcohol use as more difficult to discuss (19% versus 15%) and diagnose (56% versus 70%) than depression, and were more comfortable discussing sexual practices than alcohol (32% versus 22%). They were more likely to screen boys than girls (male PCPs were even more likely: 23% versus 6% [p < 0.0001]). Self-reported screening rates were far higher than electronic medical record (EMR)-documented rates for all substances. Experience, specialty, and recent AOD training (all p < 0.05) predicted self-reported rates; only patient age predicted actual rates. Organizational approaches (e.g., EMR tools and workflow) may matter more than PCP or patient characteristics in determining screening. Respondents reported that SBIRT was highly feasible and that it improved care; more (77) teens receiving SBIRT were referred for further assessment than those receiving usual care, and specialty treatment initiation increased from 8.73% to 12% (p < 0.0001). Organizational factors, lack of training, and discomfort with screening may impact adolescent screening and intervention. Integrated models of care for adolescent behavioral health care should be considered.

